# Identification of prognostic biomarkers related to retinoic acid metabolism in gliomas and analysis of their impact on the immune microenvironment

**DOI:** 10.1097/MD.0000000000039836

**Published:** 2024-10-11

**Authors:** Suiyun Xu, Gao Yang, Fangli Xu, Yuting Yang, Juan Wang

**Affiliations:** a Department of Neurosurgery, The Second Affiliated Hospital of Xi’an, Jiaotong University, Xi’an, China; b Department of Radiotherapy, The Second Affiliated Hospital of Xi’an, Jiaotong University, Xi’an, China; c Department of Neurosurgery, Xijing Hospital, Airforce Military Medical University (Fourth Military Medical University), Xi’an, China.

**Keywords:** biomarkers, glioma, immune cells, metabolism, prognostic signature, retinoic acid

## Abstract

Glioma is a primary tumor of the central nervous system. Numerous investigations have demonstrated that retinoic acid (RA) signaling plays an important role in glioblastoma. This research aimed to develop a RA metabolism–related gene signature associated with glioma. The RA metabolism–related differentially expressed genes were obtained through differential analysis of RA metabolism–related genes in GSE4290. The univariate Cox and least absolute shrinkage and selection operator regression analysis were adopted to build a RA metabolism–related glioma prognostic signature. We further conducted immune feature estimation and functional enrichment analysis between 2 risk subgroups. Finally, the potential drug-targeting prognostic genes were predicted through the DrugBank database. A sum of 10 RA metabolism–related differentially expressed genes between normal and tumor groups were identified. Then, a RA metabolism–related prognostic signature was built based on the 7 prognostic genes (*ADH4*, *DHRS3*, *DHRS9*, *LRAT*, *RDH10*, *RDH12*, and *RDH5*). Glioma patients were separated into 2 risk subgroups (low-risk vs high-risk) based on the median value of the risk score. We found that monocytes were negatively correlated with *DHRS9*, while activated naive CD4+T cell was positively correlated with *RDH10*. These prognostic genes participated in some immune-related processes, such as “B cell–mediated immunity.” Finally, 4 drugs targeting *DHRS3*, *LRAT*, and *RDH12* were predicted, including vitamin A, nicotinamide adenine dinucleotide, ethanol, and cyclohexylformamide. The prognostic signature comprised of *ADH4*, *DHRS3*, *DHRS9*, *LRAT*, *RDH10*, *RDH12*, and *RDH5* based on RA metabolism was established, which provided a theoretical basis and reference value for the research of glioma.

## 1. Introduction

Glioma is one of the most common primary cranial tumors in adults, with an increasing incidence in recent years.^[1]^ Currently, the main treatment option is surgery plus postoperative combined temozolomide with simultaneous radiotherapy, and some emerging therapies targeting key signaling pathways have also been reported to have good results.^[2–4]^ However, most patients with gliomas currently have a poor prognosis due to limited immune cell infiltration and low immunogenicity in the tumor tissue.^[5]^ Tumors can be affected by inflammation in 2 ways: while acute inflammation can have antitumor effects, chronic inflammation can also lead to treatment resistance, metastasis, and tumor growth through processes such as immune cell proliferation in the microenvironment.^[6]^ Therefore, exploring novel biomarkers for immunotherapy optimization in glioma individuals was urgent.

Retinoic acid (RA) is an active derivative of vitamin A, which played an important role in the immune system, cell proliferation and differentiation, and embryonic development by activating nuclear receptors to initiate and regulate gene expression.^[7–9]^ According to recent studies, the pathogenesis of gliomas has been linked to the involvement of RA, which may have a significant impact on the prognosis of patients with gliomas.^[10,11]^ Recent studies in animals have shown that RA could promote the proliferation of neurogenesis in the cortex and hippocampus.^[12]^ Some scholars have suggested that the prognosis of patients with glioblastoma might be associated with RA-related pathways.^[13]^ Some studies have also shown that all-*trans* RA may directly or indirectly inhibit the proliferation of glioma cells.^[14–16]^ These studies collectively demonstrate that RA-related pathways might hold a significant role in the development of glioma. Targeting the metabolism of RA may contribute to the prognosis and treatment of glioma patients. Therefore, we want to identify RA metabolism–related biomarkers in glioma patients, analyze their prognostic value, construct a prognostic model, and perform immunocorrelation analysis, to provide a new direction in the search for new prognostic markers for glioma.

## 2. Materials and methods

### 2.1. Data acquisition

The RNA-sequencing information of glioma was collected from The Cancer Genome Atlas (TCGA) database, which included 151 glioblastoma multiforme samples and 450 low-grade glioma samples with complete survival information. The GSE4290 dataset, including 157 glioma samples and 23 normal tissue samples, was downloaded from the Gene Expression Omnibus database. Data from 325 glioma samples with complete survival information were used for validation set, which was retrieved from the Chinese Glioma Genome Atlas (CGGA) database. A total of 19 RA metabolism–related genes (RAMGs), including *CYP2C9*, *DHRS3*, *ADH1B*, *CYP3A4*, *UGT2B7*, *RDH8*, *RDH11*, *ADH5*, *RPE65*, *RDH5*, *DHRS9*, *ALDH1A1*, *RDH16*, *ADH4*, *CYP2A6*, *RDH12*, *ALDH1A2*, *RDH10*, and *LRAT* were derived from previous research.^[17]^

### 2.2. RAMG expression comparison

The rank–sum test was yielded to analyze the difference of RAMG expression between the tumor and normal groups in the GSE4290 dataset. The RA metabolism–related differentially expressed genes (RAM-DEGs) were filtrated to the following analysis.

### 2.3. Construction of prognostic model

Based on RAM-DEGs, univariate Cox regression analysis and least absolute shrinkage and selection operator (LASSO) regression analysis were yielded to obtain prognostic genes in the training cohort (TCGA) dataset. Survival and survival time were used as components of the risk score, which was calculated by the risk model formula: risk score = ∑ n (coefficient[genei] × i = 1 expr [genei]). Glioma patients with survival information were classified into 2 risk subgroups (low-risk vs high-risk) based on the median value of the risk score for each sample. The Karnofsky score is commonly used to assess a patient’s prognosis and treatment outcome. It is based on factors such as a patient’s ability to perform daily activities, work, and self-care, and ranges from 0 (dead) to 100 (completely normal). Higher Karnofsky scores usually mean that patients have better health and quality of life. The reliability of the risk score and prognostic model was assessed by Kaplan–Meier analysis and receiver operating characteristic curve (1, 2, and 3 years), respectively. At the same time, the CGGA dataset was regarded as an external verification dataset for the risk model.

### 2.4. Identification of independent prognostic factors

Race, age, gender, Histology grade, radiation, and Karnofsky score were enrolled in the univariate Cox analysis. The cox.zph function of the “survival” package was used to perform a proportional hazards hypothesis test on significant factors to determine whether these factors satisfied the multivariate Cox regression model. Multivariate analysis was performed to screen for independent prognostic factors. The nomogram containing independent prognostic factors was drawn to predict 1-, 3-, and 5-year survival probability of glioma patients. Evaluation of the predictive effect was done by the calibration curve. In addition, risk scores between subgroups with different clinical characteristics were compared via the rank–sum test. Meanwhile, the expression of prognostic genes in different grades of tumors and different subtypes of clinical features was analyzed.

### 2.5. Immune feature estimation analysis

The relative abundance of 22 immune cells infiltrated in different risk groups (high vs low) was determined using the Cell-type Identification By Estimating Relative Subsets Of RNA Transcripts algorithm.^[18]^ Subsequently, the differences of these immune cells between 2 risk subgroups were compared. We performed the Spearman method to analyze the correlations of prognostic genes with differential immune cells. The differences in the expression levels of 8 immune checkpoint molecules (CD274, CTLA4, LAG3, LGALS9, TIMP3, PDCD1, PDCD1LG2, and TJAP1) between 2 risk groups were compared. The “estimate” package^[19]^ was applied to obtain and compare the immune, stromal, and Estimation of STromal and Immune cells in MAlignant Tumour tissues using Expression data scores of tumor tissues in 2 risk subgroups.

### 2.6. Functional enrichment analysis

Based on difference analysis of high and low expression groups of prognostic genes, the “clusterProfiler” package^[20]^ was performed for gene set enrichment analysis, which explored the potential pathways of prognostic genes. The reference gene set was “c5.go.bp.v7.5.symbols.gmt” in the Molecular Signatures Database, and false discovery rate < 0.05 was considered meaningful. Besides that, GeneMANIA was applied to construct the interaction network of prognostic genes. The differences in pathways between the high- and low-risk groups were compared using the “Gene Set Variation Analysis” package. The reference gene set was “ c2.cp.kegg.v7.4.symbols” in the Molecular Signatures Database.

### 2.7. Potential drug prediction analysis

Through the DrugBank database, the targeting medications were found in order to investigate prospective therapeutic treatments for prognostic genes in glioma.

## 3. Results

### 3.1. RAM-DEG–based gene signature for glioma outcome prediction

**Figure 1.**
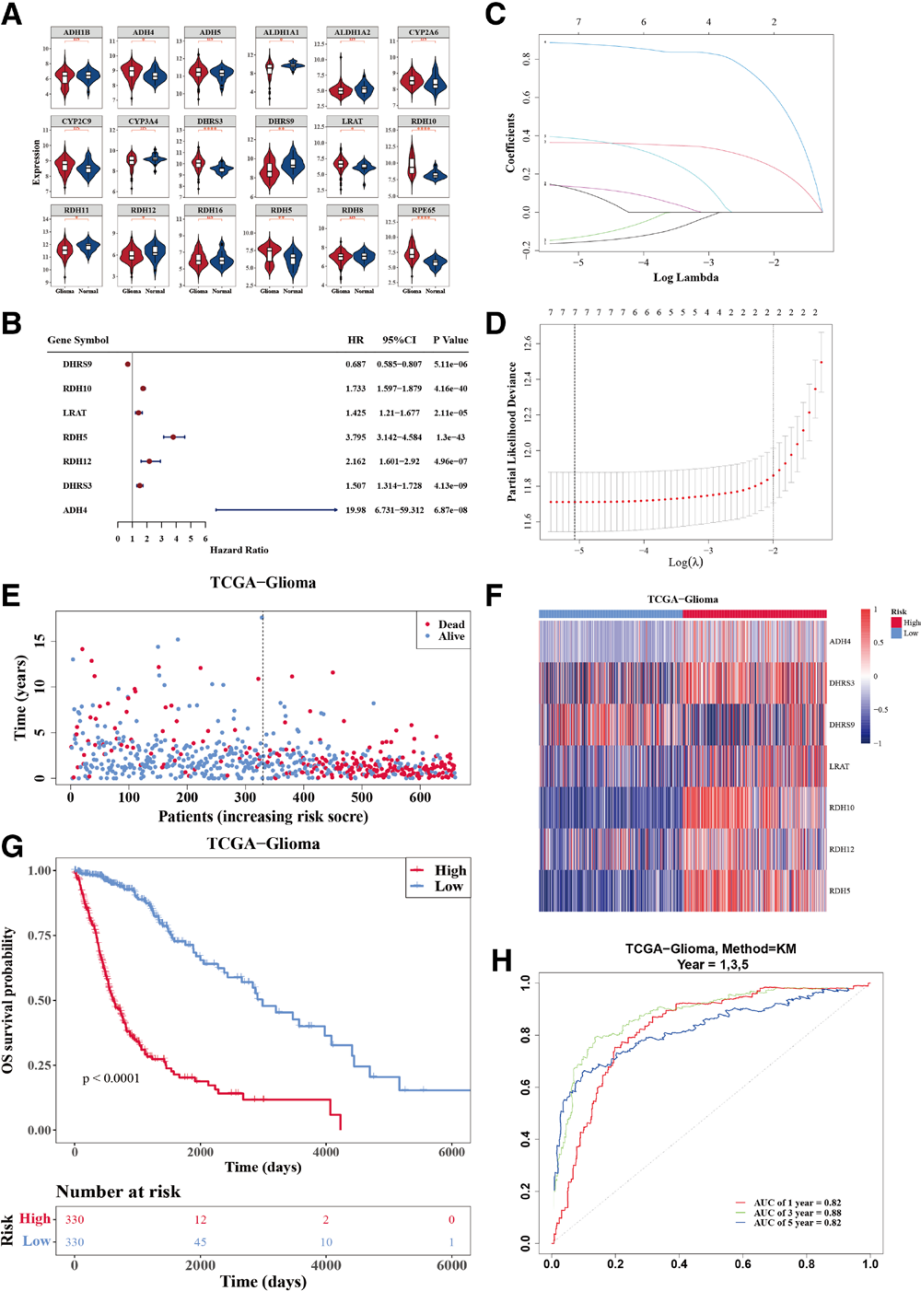
Identification of RAM-DEGs and construction of risk prognostic model. (A) RAM-DEGs between glioma and normal groups. (B–D) Univariate Cox regression analysis and LASSO regression analysis to obtain 7 prognostic genes in the training cohort. (E) Two risk groups (high and low) of glioma patients categorized based on the median value of the risk score from the risk prognostic model. (F) Expressions of 7 prognosis genes in 2 risk groups. (G) Survival analysis of 2 risk groups. (H) ROC curve of the risk prognostic model (1, 2, and 3 years). AUC = area under the curve, CI = confidence interval, HR = hazard ratio, KM = Kaplan-Meier, LASSO = least absolute shrinkage and selection operator, OS = overall survival, RAM-DEG = retinoic acid metabolism–related differentially expressed genes, ROC = receiver operating characteristic, TCGA = The Cancer Genome Atlas.

There were 10 RAM-DEGs between tumor and normal groups, including *ADH4*, *ALDH1A1*, *DHRS3*, *DHRS9*, *LRAT*, *RDH10*, *RDH11*, *RDH12*, *RDH5*, and *RPE65* (Fig. 1A). Univariate regression analysis identified 7 significant genes (hazard ratio ≠ 1; *P* < .05) in the training cohort (Fig. 1B). To further screen the key genes, LASSO regression analysis was conducted on these genes (Fig. 1C and 1D). Ultimately, *ADH4*, *DHRS3*, *DHRS9*, *LRAT*, *RDH10*, *RDH12*, and *RDH5* were selected as prognostic genes for building an RA metabolism–related prognostic signature with glioma. Then, glioma patients were categorized into 2 risk groups (high and low) (Fig. 1E). *ADH4*, *DHRS3*, *LRAT*, *RDH10*, *RDH12*, and *RDH5* were higher expressed in the high-glioma-risk group while *DHRS9* was lower expressed (Fig. 1F). Notably, we found significant survival differences between 2 risk groups, and patients in the high-glioma-risk group had poor survival rates (Fig. 1G). To further evaluate the reliability of the model, the area under the curve value of the model in forecasting 1-, 3-, 5-year survival of glioma patients was 0.82, 0.88, and 0.82 in the training set, indicating the risk model could forecast the survival status of glioma patients (Fig. 1H).

**Figure 2.**
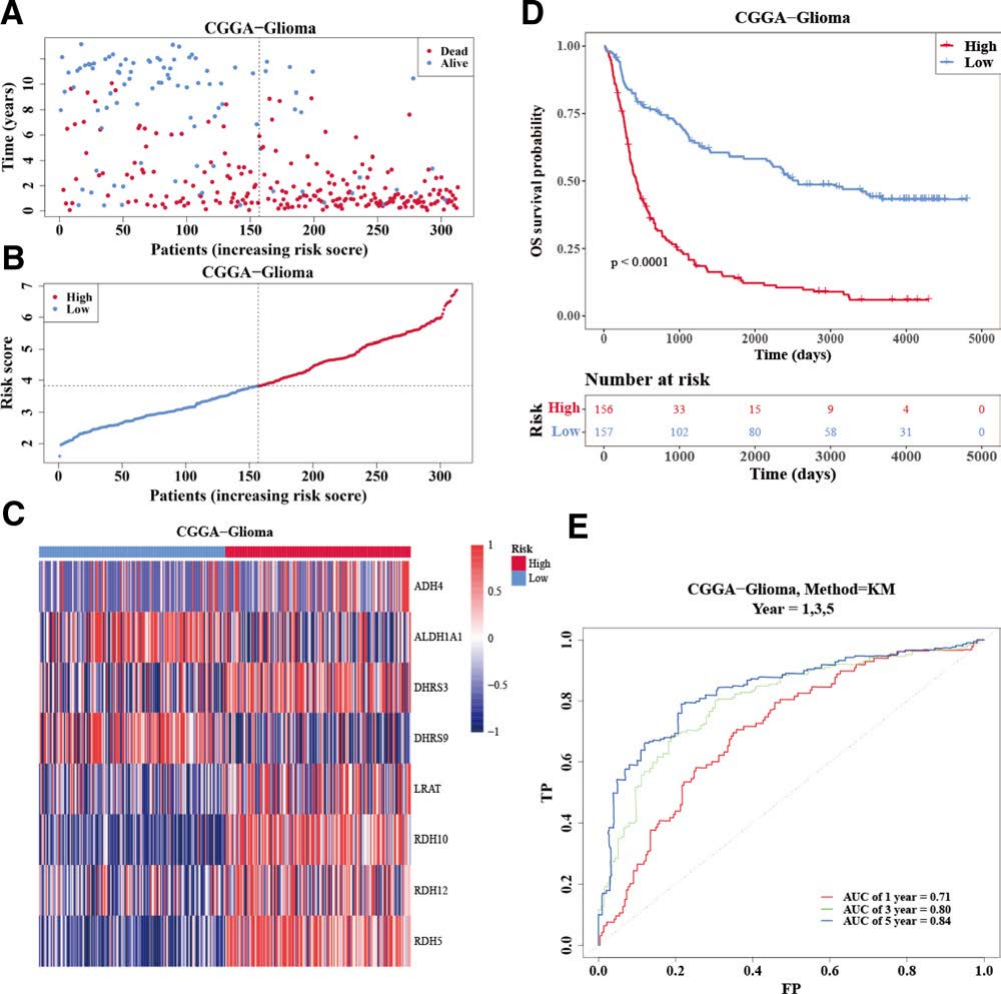
External validation of the reliability of the risk score and prognostic model. (A and B) Two risk groups (high and low) of glioma patients categorized based on the median value of the risk score from the risk prognostic model. (C) Expressions of 7 prognosis genes in 2 risk groups. (D) Survival analysis of 2 risk groups. (E) ROC curve of the risk prognostic model (1, 2, and 3 years). AUC = area under the curve, CGGA = Chinese Glioma Genome Atlas, FP = false positive, KM = Kaplan-Meier, OS = overall survival, ROC = receiver operating characteristic, TP = true positive.

Next, we further validated the risk model in the external validation datasets (CGGA). Consistent with the results generated from the training set (Fig. 2A–2C), glioma patients (high-risk group) had bad rate of survivors (Fig. 2D). Area under the curve of 1, 3, and 5 years was basically >0.60 (Fig. 2E). These results showed that the RA metabolism–related gene signature exhibited appropriate predicting performance in the validation set.

### 3.2. Independent prognostic analysis for glioma patients and the nomogram based on the prognostic independent factors

**Figure 3.**
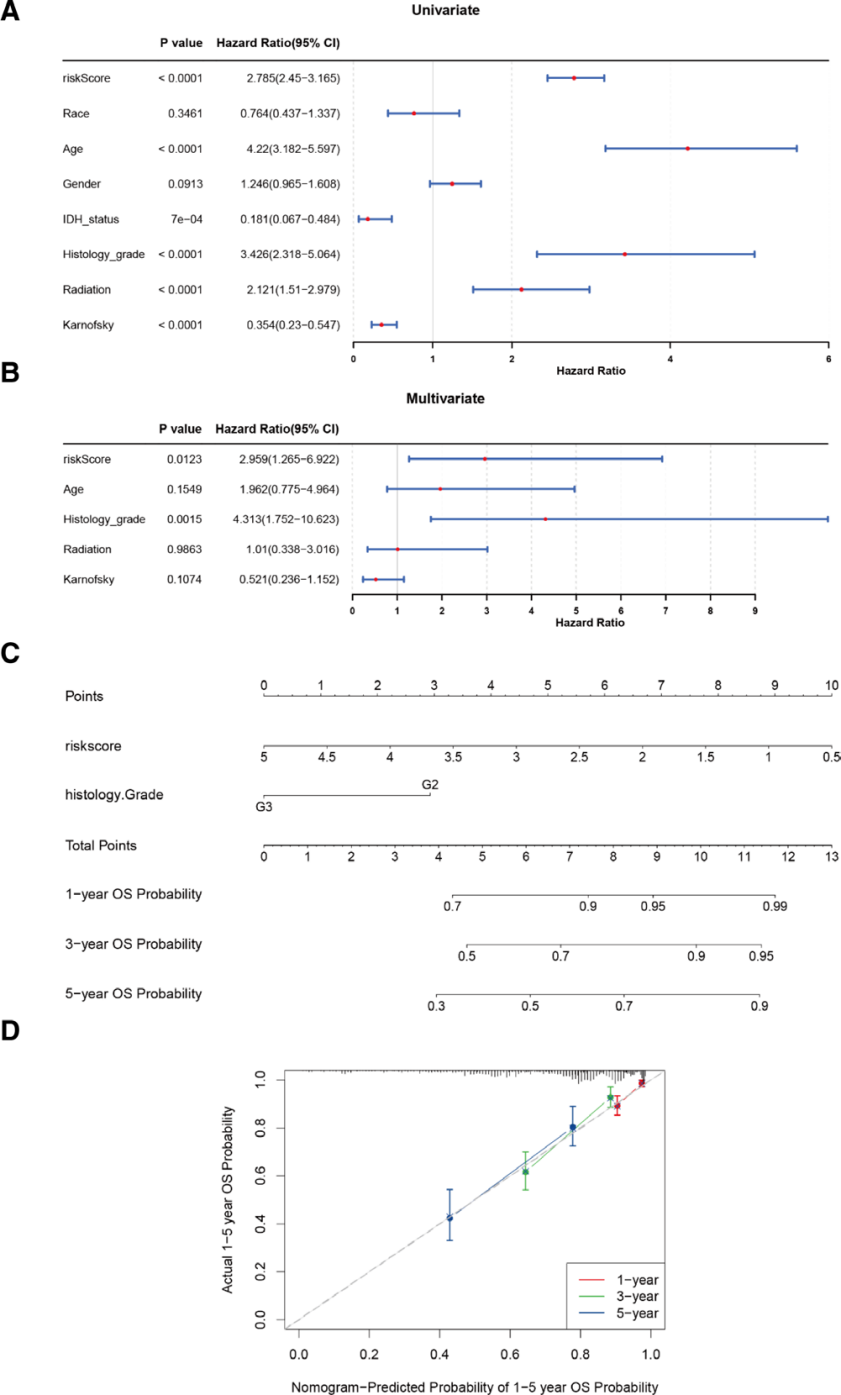
Independent prognostic analysis for glioma patients and the nomogram based on the prognostic independent factors. (A) Results of the univariate Cox analyses. (B) Results of the multivariate Cox analyses. (C) Nomogram based on the prognostic independent factors. (D) Calibration curve of the nomogram. CI = confidence interval, IDH = isocitric dehydrogenase, OS = overall survival.

The results of univariate Cox analyses suggested that risk score, age, histology grade, radiation, and Karnofsky score were prognostic factors (*P* < .05) (Fig. 3A). Proportional hazards hypothesis test analysis showed that only risk score and histology grade met the multivariate Cox regression model. Furthermore, these 2 factors were considered as prognostic independent factors in glioma (Fig. 3B). The nomogram containing 1-, 3-, 5-year survival rates was generated, including age, race, histology grade and risk score (Fig. 3C). The calibration curve proved that the feasibility of the nomogram was effective (Fig. 3D).

### 3.3. Association between clinicopathological features and risk signature

**Figure 4.**
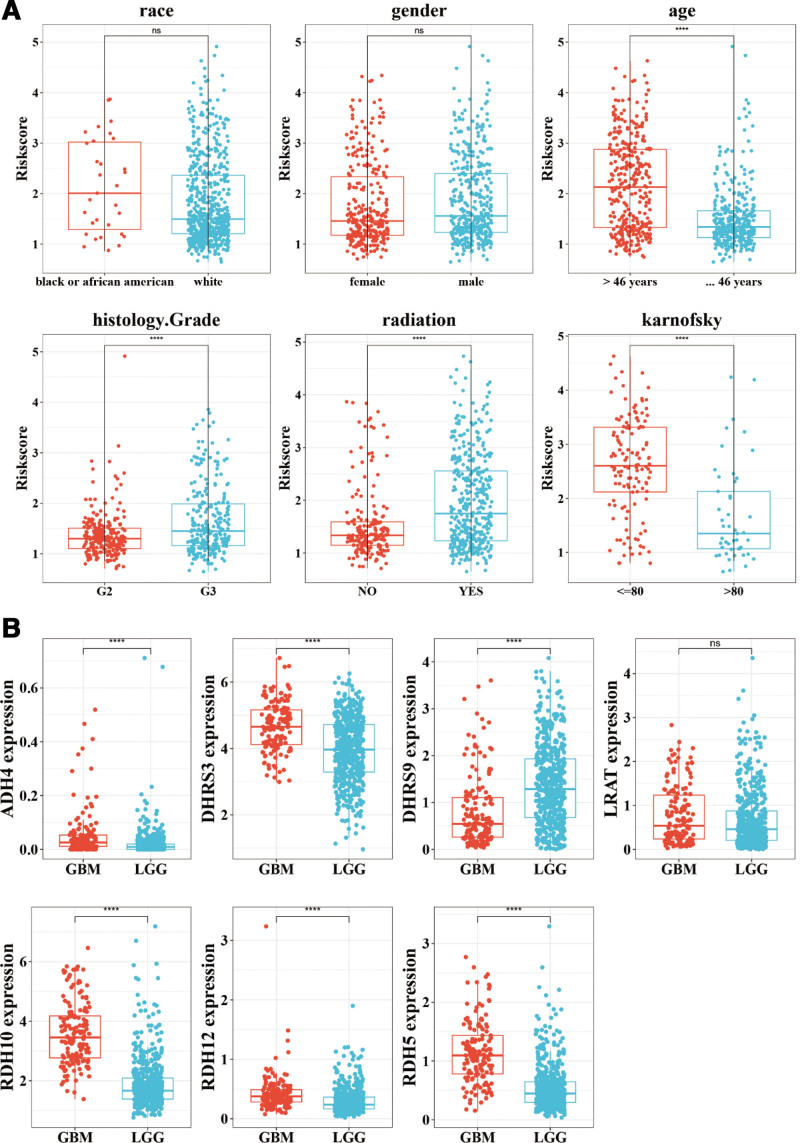
Association between clinicopathological features and risk signature. (A) Differences in risk scores among race, gender, age, histology grade, radiation, and karnofsky. (B) Expression levels of 7 prognostic genes between GBM and LGG. GBM = glioblastoma multiforme, LGG = low-grade glioma.

We found that there were significant differences in risk scores among age, histology grade, radiation, and Karnofsky score (Fig. 4A). Meanwhile, the expression levels of these 7 prognostic genes were significantly different between glioblastoma multiforme and low-grade glioma (Fig. 4B). The expression levels of *ADH4*, *DHRS9*, *LRAT*, *RDH10*, and *RDH5* were significantly different in age and histology grade (Figure S1, Supplemental Digital Content, http://links.lww.com/MD/N618).

### 3.4. Immune infiltration analysis associated with prognostic genes

**Figure 5.**
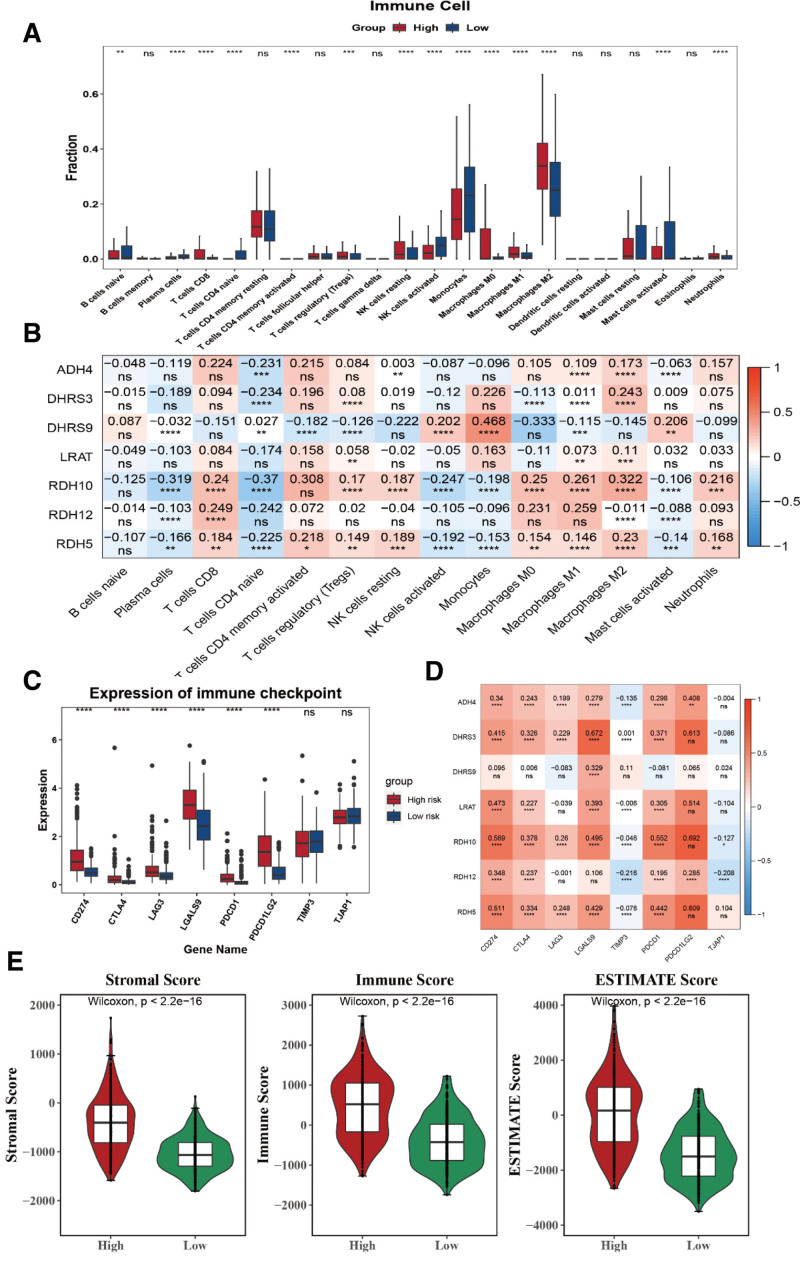
The immune infiltration analysis associated with prognostic genes. (A) Differences in immune cell infiltration between the high- and low-risk groups. (B) Correlation between the 7 prognostic genes and immune cells. (C) Expression levels of 8 immune checkpoint molecules between the high- and low-risk groups. (D) Correlation between immune checkpoints and prognostic genes. (E) Differences in immune, stromal, and ESTIMATE scores between the high- and low-risk groups. NK = natural killer.

To explore the immune microenvironment of glioma, we analyzed the expression of tumor microenvironment cells between tumor and normal groups (Figure S2, Supplemental Digital Content, http://links.lww.com/MD/N618). There were 14 immune cell abundances that differed significantly in 2 risk groups, including naive B cells, plasma cells, CD8 T cells, and activated CD4 memory T cells (Fig. 5A). The correlation between different differential immune cells was shown in Figure S3, Supplemental Digital Content, http://links.lww.com/MD/N618. Meanwhile, the correlation analysis revealed that monocytes were negatively correlated with *DHRS9* (Spearman *R* = 0.468), while activated naive CD4 T cell was positively correlated (Spearman *R* = −0.37) (Fig. 5B). Notably, we also found that 6 immune checkpoints were significantly upregulated in the high-glioma-risk group, including CD274, CTLA4, LAG3, LGALS9, PDCD1, and PDCD1LG2 (Fig. 5C). Three prognostic genes, including *RDH5*, *RDH10*, and *DHRS3*, simultaneously have a substantial positive connection with PDCD1LG2 (Fig. 5D). Coincidentally, the immune, stromal, and Estimation of STromal and Immune cells in MAlignant Tumour tissues using Expression data scores of the high-glioma-risk group were significantly higher than those of the low-glioma-risk group (Fig. 5E).

### 3.5. Functional pathways of biomarkers

**Figure 6.**
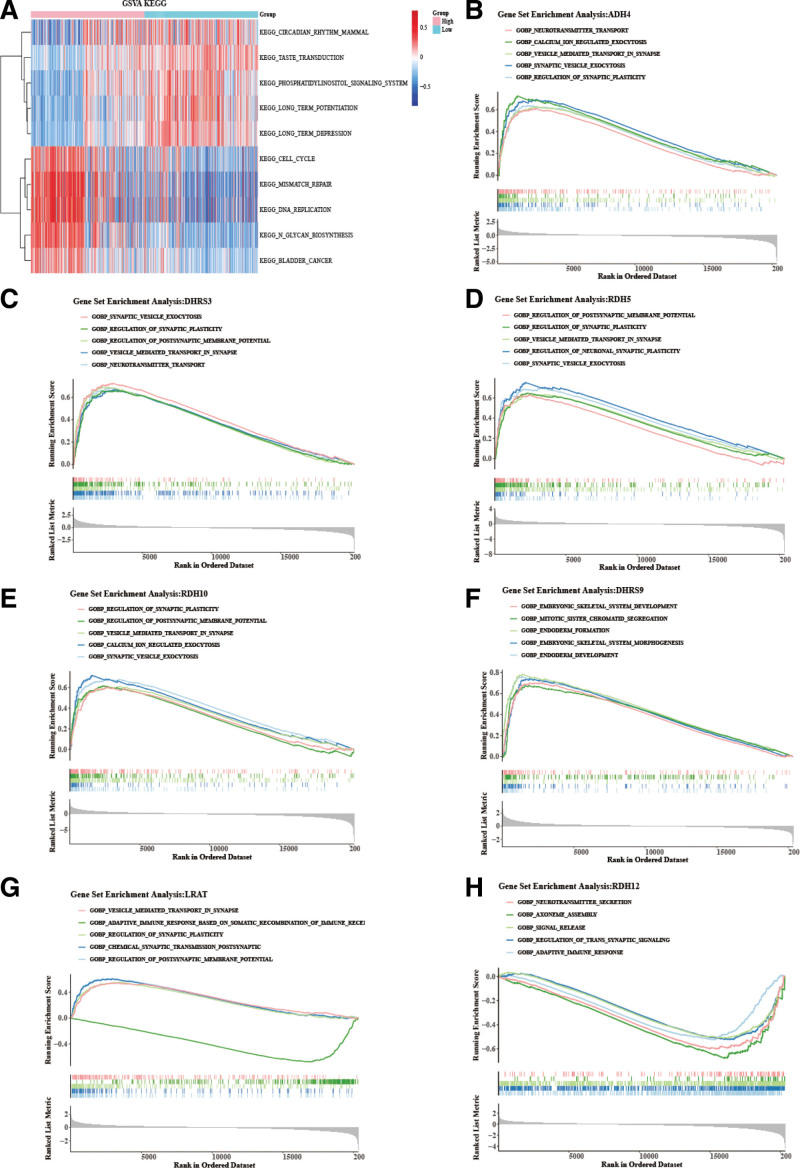
The functional pathways of biomarkers. (A) Differences in pathways between 2 risk groups. (B–H) Important biological processes involved in 7 prognostic genes. GSVA = gene set variation analysis, KEGG = Kyoto Encyclopedia of Genes and Genomes.

To further investigate the biological pathways for RA metabolism–related prognostic model, we compared the differences in pathways between 2 risk groups. The results suggested that “DNA replication,” “cell cycle,” and “mismatch repatr” were enriched in high-glioma-risk groups, while “phosphatidylinositol ” was enriched in low-glioma-risk groups (Fig. 6A). We continue to explore the important biological processes involved in those 7 prognostic genes. The analysis demonstrated that *ADH4* and *DHRS3* simultaneously participated in the “regulation of postsynaptic membrane potential” and “regulation of synaptic plasticity” processes (Fig. 6B and 6C). *RDH5* and *RDH10* were related to “synaptic vesicle exocytosis” processes (Fig. 6D and 6E). *DHRS9*, *LRAT*, and *RDH12* were associated with some immune-related processes, such as “humoral immune response” and “B cell–mediated immunity”(Fig. 6F–6H).

### 3.6. Drug analysis of prognostic genes

**Figure 7.**
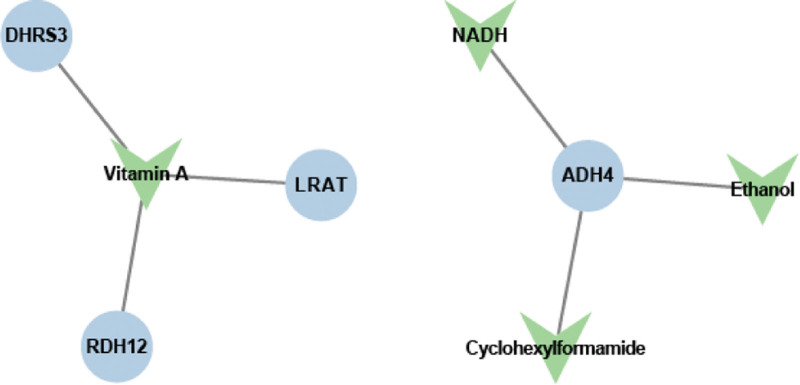
Drug analysis of prognostic genes.

In order to discover potential targeting drugs, we predicted the drugs targeting 7 prognostic genes through the DrugBank database. A total of 4 drugs of targeting *DHRS3*, *LRAT*, and *RDH12* were obtained, including vitamin A, NADH, ethanol, and cyclohexylformamide (Fig. 7).

## 4. Discussion

RAs, a group of vitamin A–related compounds, have been studied in a variety of cancers for their ability to induce terminal differentiation, promote apoptosis, and arrest cell growth.^[21]^ RA has long been considered a promising area for cancer prevention and chemotherapy. RA and its derivatives have been shown to inhibit growth and induce apoptosis in a variety of epithelial cancer cells, may target undifferentiated stem-like cells to inhibit tumor growth, and have been successfully used in the treatment of acute promyelocytic leukemia.^[22–26]^ The role of RA in glioma has been gradually discovered in recent years. Thus, in this study, we identified 7 RA-related biomarkers associated with glioma prognosis through the use of bioinformatics methods. Moreover, we delved into the potential mechanisms underlying the action of the prognostic biomarkers within the immune microenvironment.

Based on the GSE4290 dataset and the 19 RAMGs, we identified 10 RAM-DEGs between the tumor and normal groups. Then, by univariate Cox regression analysis and LASSO regression analysis, 7 genes were selected as prognostic genes: *ADH4*, *DHRS3*, *DHRS9*, *LRAT*, *RDH10*, *RDH12*, and *RDH5*. Then, these genes were used for the construction of a risk model. Through the data of the training cohort (TCGA) and test cohort (CGGA), we conducted the evaluation and validation of the risk model and found that the model has good predictive efficacy, which might play as an independent biomarker for the prognostic evaluation of glioma. To further investigate the prognostic implications of clinical and pathological characteristics along with risk models, we incorporated factors such as race, age, gender, histology grade, radiation, and Karnofsky score into a risk model along with risk scores for single-factor and multifactor Cox independent prognostic analyses in the glioblastoma samples from the training dataset. The results revealed that risk score and histology grade serve as good independent prognostic factors. Subsequently, we constructed a nomogram to predict the prognosis of glioblastoma patients using these 2 factors and validated that it possesses good predictive capabilities.

Ethanol dehydrogenase 4 (ADH4) was a member of the ADH family. ADH4 was associated with diseases like Parkinson and exhibits dysregulated expression in various tumors, such as lung cancer and hepatocellular carcinoma.^[27–29]^ As a member of the short-chain dehydrogenase/reductase (SDR) family, DHRS3 encoded an enzyme that plays a crucial role in the metabolism of retinol (vitamin A). Furthermore, studies have suggested its potential involvement in the development and progression of various cancers, including gastric and thyroid cancer.^[30,31]^ DHRS9, a member of the SDR family, is thought to be a promising target for preventing the formation of malignancies like colon cancer since it has been reported in multiple studies to play a critical role in the development and progression of various tumors.^[32–34]^ Lecithin retinol acyltransferase (LRAT) served as the primary enzyme responsible for facilitating the esterification of vitamin A (all-*trans* retinol). Previous research has indicated that its expression might be linked to various tumors, including bladder cancer, colon cancer, and melanoma, and it may be utilized to exhibit antitumor capabilities.^[35–37]^ Retinol dehydrogenase 10 (RDH10) belonged to the SDR family and exhibits retinol oxidoreductase activity. Numerous studies have demonstrated that RDH10 was linked to the development and progression of various tumors, including liver cancer and prostate cancer.^[38,39]^ Of particular interest, research suggests a potential connection between RDH10 and glioma patient prognosis, as well as a role in the proliferation, survival, and metastasis of glioma cells, which aligns with the findings of our study.^[40,41]^ Retinol dehydrogenase 12 (RDH12) was a NADPH-dependent retinol reductase expressed in tissues such as photoreceptors, skin, and kidneys.^[42]^ In addition to its role in retinal diseases, RDH12 was believed to be potentially associated with tumors such as cervical cancer, gastric cancer, and head and neck squamous cell carcinoma.^[43–45]^ Retinol dehydrogenase 5 (RDH5) was a key enzyme involved in the oxidation of 11-*cis*-retinol and the metabolism of RA/all-trans retinoic acid. In addition to its association with fundus albipunctatus, some studies have suggested that it may be potentially related to tumors such as hepatocellular carcinoma and gastric cancer.^[46–48]^

To date, except for RDH10, no comprehensive studies have established a link between the remaining 6 genes and glioma. Consequently, we postulate that these 7 genes might influence the prognosis of glioma patients by interacting with the immune microenvironment within the tumor. By analyzing the differences in immune cell infiltration between high- and low-risk groups, we observed a significant disparity in 14 types of immune cells, such as naive B cells, plasma cells, CD8 T cells, and activated CD4 memory T cells. Then, by conducting a correlation analysis of prognostic genes and differential immune cells, we discovered that monocytes were negatively correlated with DHRS9 and activated naive CD4 T cell was positively correlated with RDH10. Previous studies have demonstrated that DHRS9 could modulate the immune response by influencing monocytes and macrophages, while RDH10 could serve as a crucial molecule in regulating T-cell differentiation and plays a significant role in tumor immunity.^[49,50]^ These findings align with our study results. After that, we found that 6 immune checkpoints (CD274, CTLA4, LAG3, LGALS9, PDCD1, and PDCD1LG2) were significantly upregulated in the high-glioma-risk group, while RDH5, RDH10 and DHRS3 simultaneously have a substantial positive connection with PDCD1LG2. A few studies have suggested a correlation between PDCD1LG2 expression and prognosis in glioma.^[51]^ Combining our research findings, this provides a new direction for the study of tumor microenvironment in glioblastoma. Lastly, we predicted potential drugs that may interact with prognostic genes, including vitamin A, NADH, ethanol and cyclohexylformamide, which offers a new research direction for subsequent drug therapy in glioblastoma.

## 5. Conclusion

This study utilized a comprehensive suite of bioinformatics methods to establish the prognostic significance of RAMGs in glioma. We identified 7 promising prognostic genes (*ADH4*, *DHRS3*, *DHRS9*, *LRAT*, *RDH10*, *RDH12*, and *RDH5*) within glioma and developed a prognostic model with significant predictive accuracy. Moreover, we delved into the potential molecular mechanisms underlying the influence of prognostic genes on glioblastoma progression. This study unlocks new avenues for uncovering the molecular mechanisms and predictive potential of glioblastoma, offering significant implications for clinical assessments, patient prognosis, and the development of innovative therapeutics. Moving forward, we will validate these findings and continue excavating the depths of this critical field.

## Author contributions

**Data curation:** Suiyun Xu.

**Formal analysis:** Suiyun Xu.

**Investigation:** Suiyun Xu, Fangli Xu.

**Writing – original draft:** Suiyun Xu.

**Writing – review & editing:** Gao Yang.

**Methodology:** Fangli Xu.

**Resources:** Fangli Xu.

**Software:** Fangli Xu.

**Validation:** Yuting Yang.

**Visualization:** Yuting Yang.

**Conceptualization:** Juan Wang.

**Supervision:** Juan Wang.

## Supplementary Material


