# Supplementary material for: Identification of prognostic biomarkers related to retinoic acid metabolism in gliomas and analysis of their impact on the immune microenvironment
Source: Medicine (Baltimore). 2024 Oct 11;103(41):e39836. doi: 10.1097/MD.0000000000039836 (PMC11479434; doi:10.1097/MD.0000000000039836)
Supplement: Supplementary file 1 [file medi-103-e39836-s001.pdf]

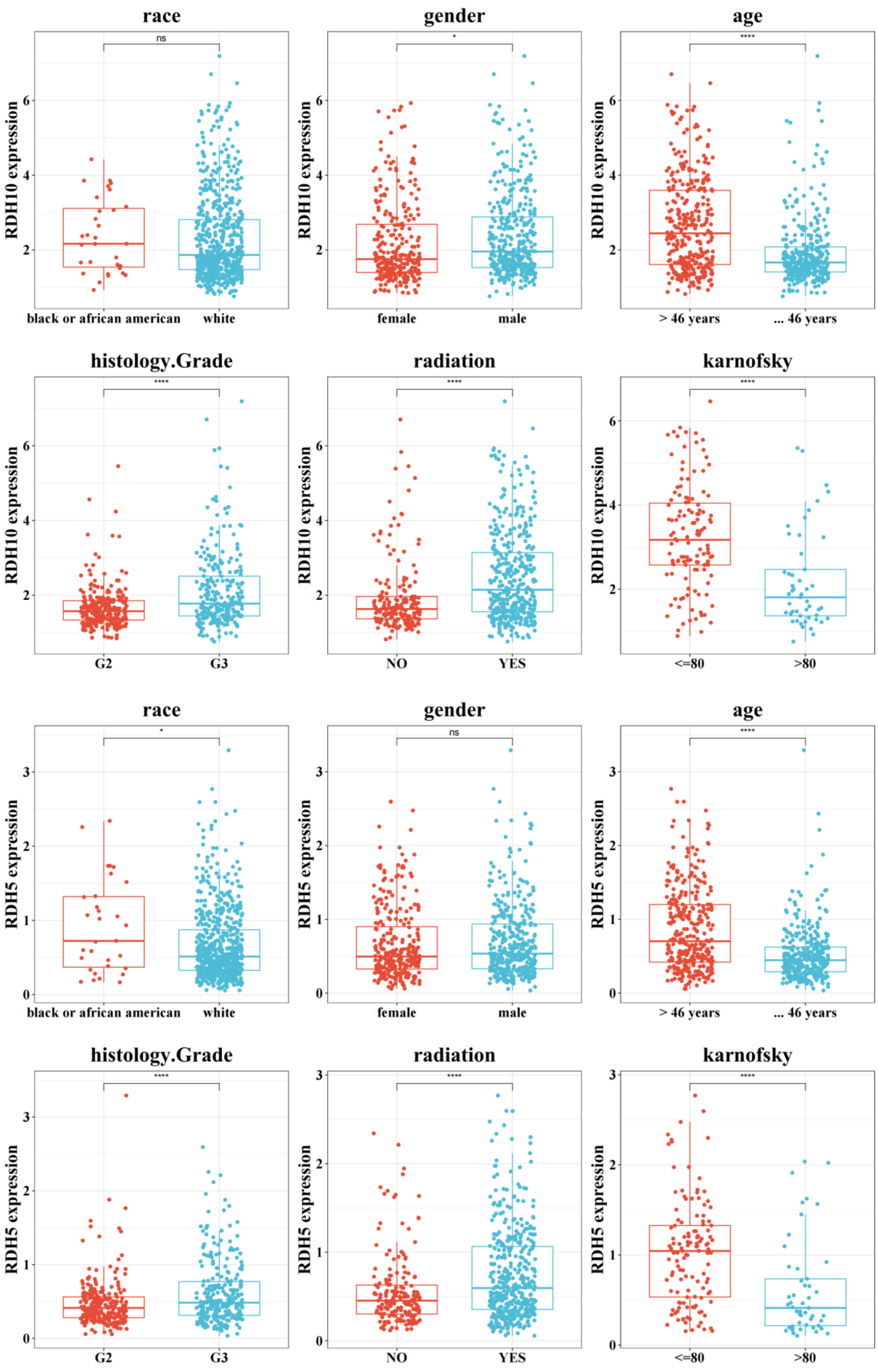
race
gender
age
histology.Grade
radiation
karnofsky
RDH10 expression
RDH5 expression
ns
*
****
black or african american
white
female
male
> 46 years
... 46 years
G2
G3
NO
YES
<=80
>80

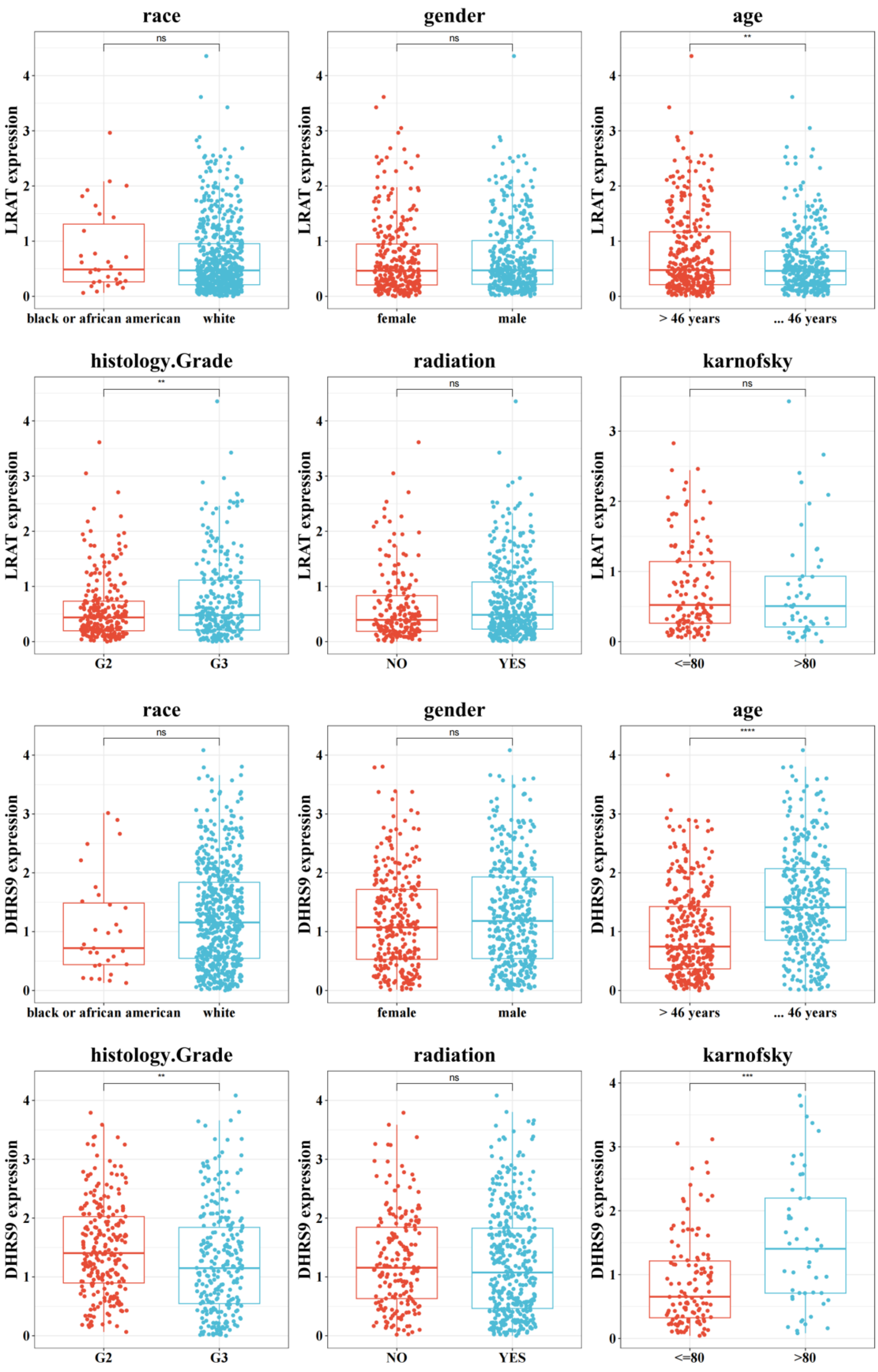

race
ns
LRAT expression
black or african american
white
gender
ns
LRAT expression
female
male
age
**
LRAT expression
> 46 years
... 46 years
histology.Grade
**
LRAT expression
G2
G3
radiation
ns
LRAT expression
NO
YES
karnofsky
ns
LRAT expression
<=80
>80
race
ns
DHRS9 expression
black or african american
white
gender
ns
DHRS9 expression
female
male
age
****
DHRS9 expression
> 46 years
... 46 years
histology.Grade
**
DHRS9 expression
G2
G3
radiation
ns
DHRS9 expression
NO
YES
karnofsky
***
DHRS9 expression
<=80
>80

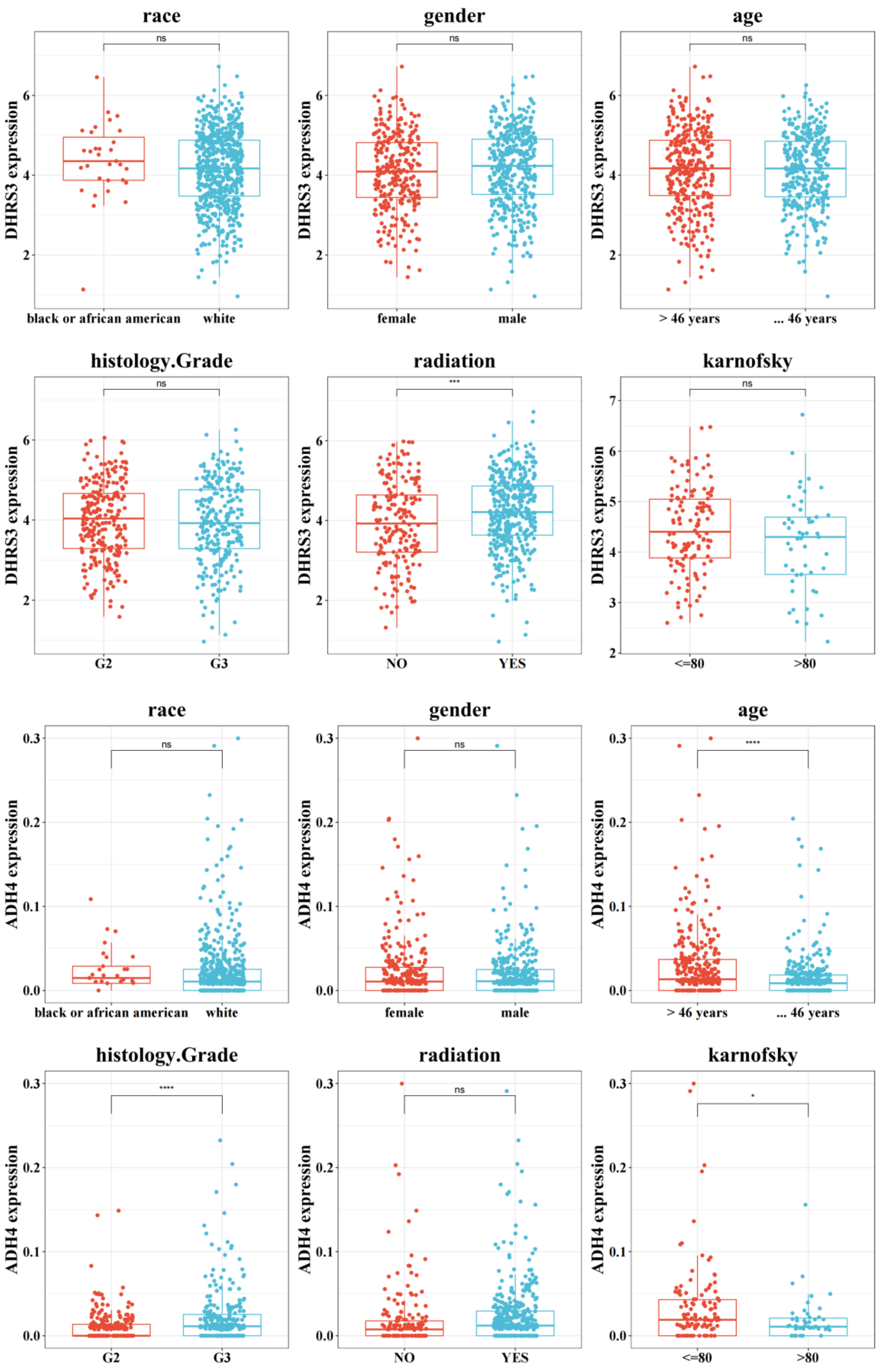
race
gender
age
DHRS3 expression
ns
ns
ns
black or african american
white
female
male
> 46 years
... 46 years
histology.Grade
radiation
karnofsky
ns
***
ns
G2
G3
NO
YES
<=80
>80
race
gender
age
ADH4 expression
ns
ns
****
black or african american
white
female
male
> 46 years
... 46 years
histology.Grade
radiation
karnofsky
****
ns
*
G2
G3
NO
YES
<=80
>80

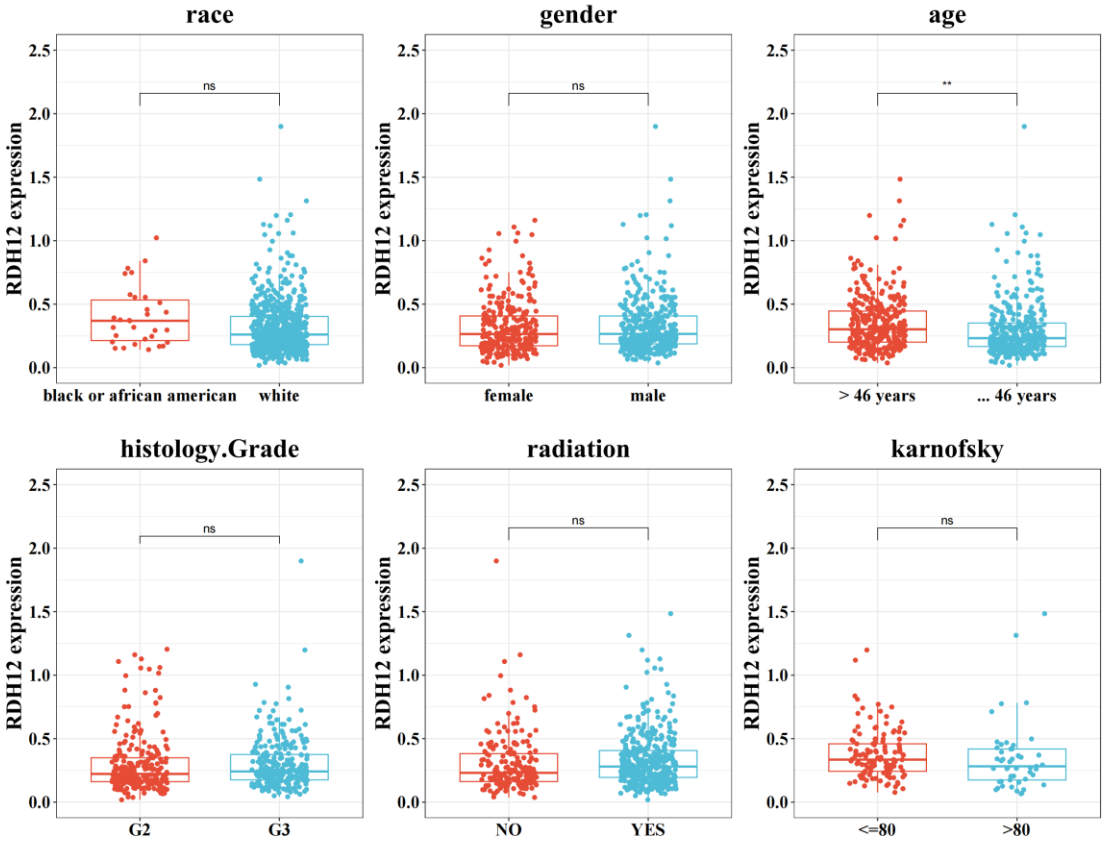

Supplement figure 1 Expression of prognostic genes in different tumor grades and clinical characteristic subtypes

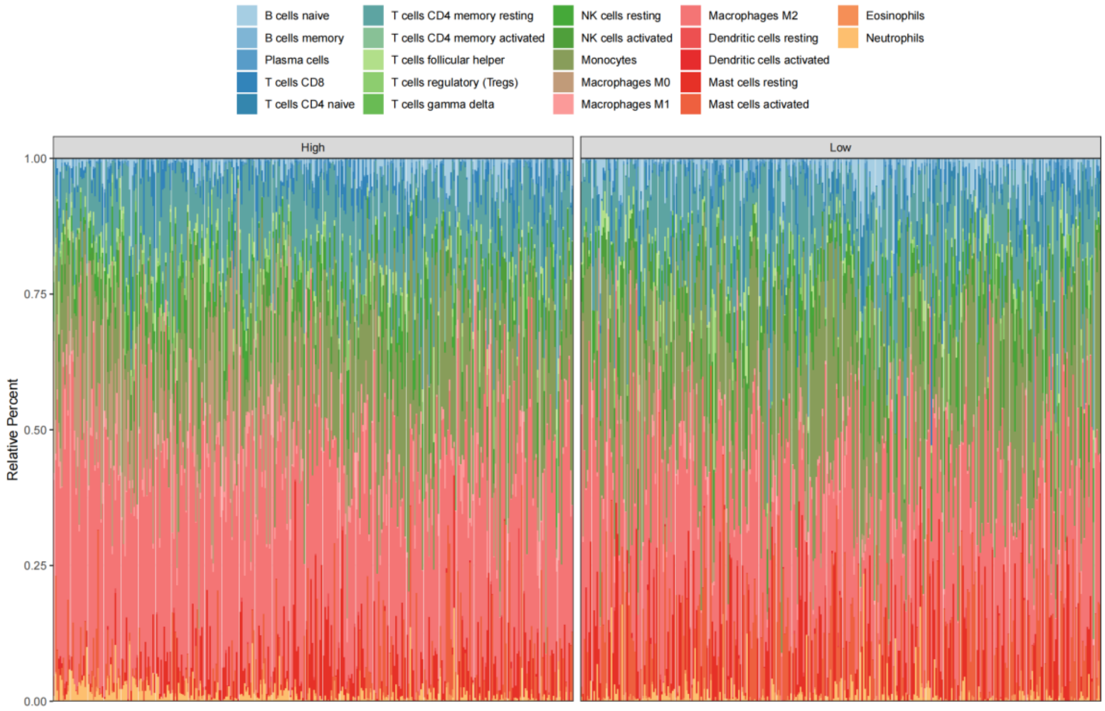

Supplemental Figure 2 the expression of tumor microenvironment cells between tumor and normal groups

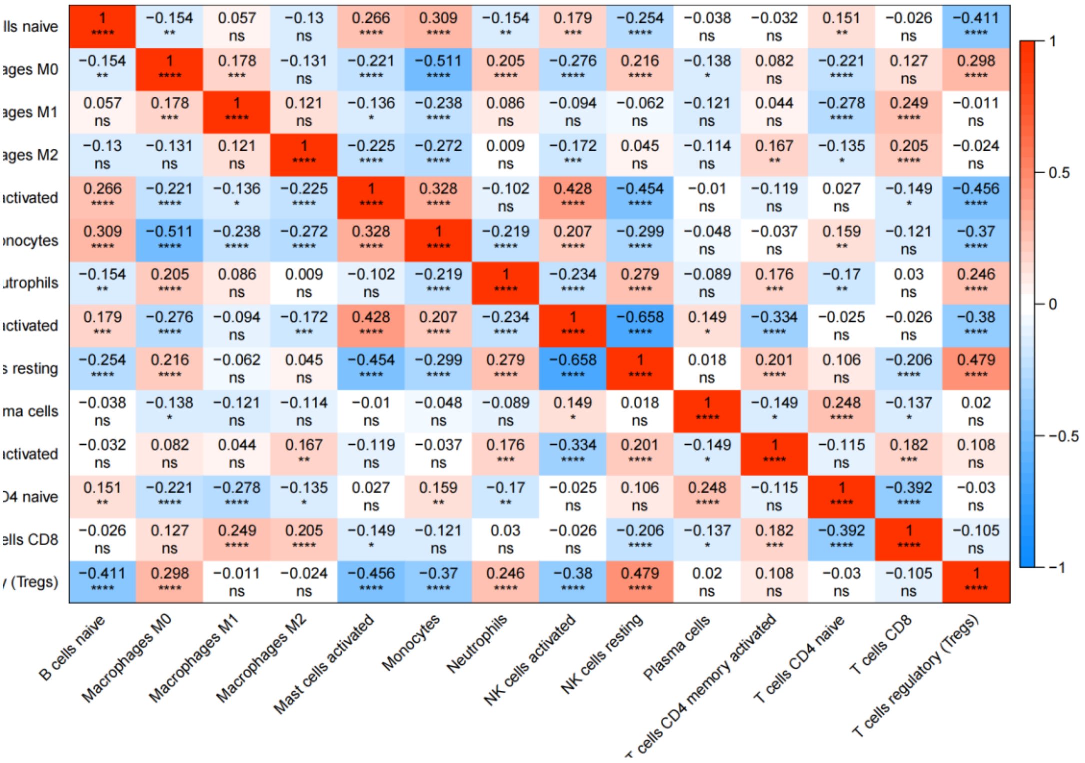

Supplemental Figure 3 The correlation between different differential immune cells
